# Chronic orofacial pain and white matter hyperintensities

**DOI:** 10.18632/aging.204584

**Published:** 2023-03-19

**Authors:** Elena Calabria, Federica Canfora, Daniela Adamo

**Affiliations:** 1 Department of Health Sciences, University Magna Graecia of Catanzaro, School of Dentistry, Catanzaro 88046, ItalyDepartment of Neurosciences, Reproductive Sciences and Dentistry, University Federico II of Naples, Naples 80131, Italy; 2 Department of Health Sciences, University Magna Graecia of Catanzaro, School of Dentistry, Catanzaro 88046, Italy

**Keywords:** white matter hyperintensities, chronic orofacial pain, burning mouth syndrome, pain, temporomandibular disorders

Chronic Orofacial Pain (COFP) is defined as an intermittent or continuous ‘pain below the orbitomeatal line, anterior to the pinnae and above the neck lasting longer than 3 months [1].

COFP is a multidimensional experience in which the structural changes of the gray and white matter of the brain in prefrontal, somatosensory, occipital areas and subcortical nuclei may be a concurrent cause of the impairment of sensory-discriminative and cognitive-affective pathways, contributing to the mechanism of pain perception [2]. The structural magnetic resonance imaging (MRI) of the brain has been widely used in patients affected by COFP in order to exclude space-occupying lesions (such as intracranial tumors and cysts), or any vascular compression of the trigeminal nerve and also to elucidate any changes in the brain function and structure which could lead to the central neuropathy [2].

**Figure 1:**
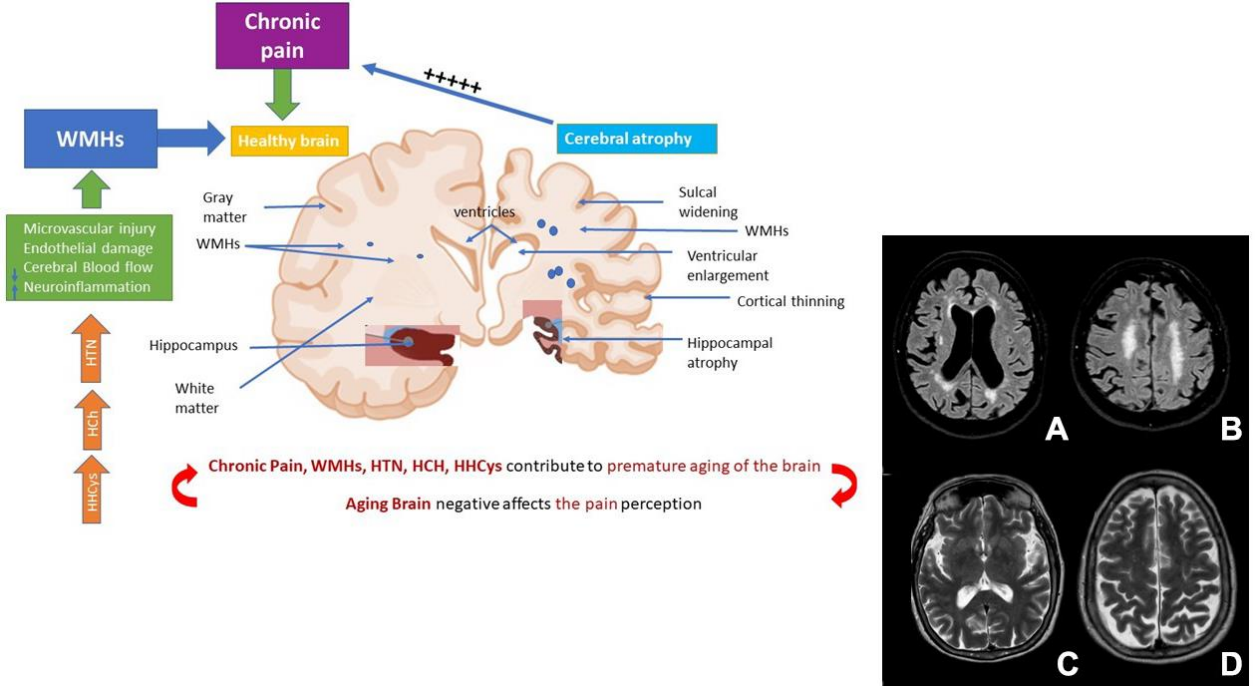
**Role of chronic pain and WHs in the premature ageing of the brain.** Abbreviations: WMHs: white matter hyperintensities; HTN: Hypertension; HCh: Hypercholesterolemia; HHCys: Hyperhomocysteinemia. (**A**, **B**) cerebrovascular disease. (**C**, **D**) brain atrophy.

In this context, white matters hyperintensities (WMHs) are the most common macrostructural brain changes occurring in later life and considered an early marker of brain frailty. They consist of patchy areas of hyperintensity signal scattered in the deep or periventricular white matter evident on brain MRI T2-weighted or Fluid Attenuation Inversion Recovery (FLAIR) sequences, as shown in Figure 1A, 1B.

The WMHs are primarily a consequence of cerebral small vessel disease (CSVD) and ageing, with a prevalence rate in elderly ranging between 39% and 96%. Notably, WMHs have been acknowledged as risk factor for stroke and premature death, and also as potential contributing factor to the pathogenesis of vascular dementia or Alzheimer’s disease. Further, WMHs influence interconnections in multiple regions of the brain, mainly from the cortical to subcortical areas, which in turn affect the descending pathway of the modulatory system of pain, playing a significant role in the amplification and processing of the pain [3].

On account of these evidences, recent advanced imaging techniques such as voxel-based morphometry (VBM), diffusion-weighted imaging (DWI) with a diffusion tensor imaging (DTI) with a fractional anisotropy (FA) and tractography have been used in patients affected by various subtypes of COFP in order to evaluate *in vivo* brain information about the white matter microstructure.

For instance, some authors have found that the onset and progression of WMHs, in trigeminal neuralgia (TN) may be attributed to pain intensity and/or pain duration. Moreover, in TN caused by neurovascular compression, a reduced microstructural integrity of white matter in thalamic-somatosensory tracts has been found only ipsilateral to the site of neurovascular conflict suggesting the potential role of white matter alterations in nociception. Specifically, in these patients demyelination occurs along the entire S1 arc in fibers indicating a global decoupling between the thalamus and S1 on the side of neurovascular compression in patients with TN [2, 4].

Furthermore, in temporomandibular disorders patients (TMD), a lower structural connectivity of white matter in corpus callosum and in dorsolateral prefrontal cortex, and a higher connectivity in the frontopolar cortex have been found through DTI analysis [4]. The structural white matter abnormalities in these areas are involved not only in pain perception but also in cognitive functions and may contribute to the cognitive impairment frequently detected in TMD patients.

Moreover, a lower FA has been found in the right internal capsule and in the cingulum suggesting a reduced axonal integrity in these areas; furthermore, a negative correlation was found between FA of the right trigeminal nerve and pain duration, and between FA of the tracts adjacent to the ventrolateral prefrontal cortex and in tracts coursing through the thalamus and internal capsule and pain intensity [2, 4].

While no studies have evaluated WMHs in persistent idiopathic facial pain and in persistent idiopathic dentoalveolar pain, a recent research has investigated the prevalence of WMHs in the brain of patients affected by Burning Mouth syndrome (BMS), a subclass of COFP. Of note, WMHs were statistically more prevalent in BMS patients compared to a pain-free control group, suggesting that the routine prescription of MRI of the brain could be useful for the WMHs detection also in other types of COFP [3, 5].

Specifically, the higher frequency of WMHs in BMS patients was mainly found in the frontal, parieto-occipital and temporal areas suggesting that the progression and the extension of WMHS in the years may have a potential role in cognitive impairment and in the development of neurodegenerative diseases. In this respect, there is a recent evidence reporting that BMS patients, at time of diagnosis, already exhibit a preclinical transitional state of cognitive impairment called “burning fog” characterized by subjective concentration difficulties, forgetfulness, mental confusion, and inability to multitask, with a decrease in global cognitive functions, elective attention, sustained attention, cognitive flexibility, working memory, and executive functions [6]. Of importance, the onset and progression of WMHs has been generally related to many cardiovascular risk factors. In this regard, hypertension (HTN), hypercholesterolemia, and hyperhomocysteinemia were found to be present in 58%, 46% and 73% respectively of BMS patients, suggesting the role of vascular comorbidity in the onset and progression of WMHs in these patients [5]. Indeed, the early onset of HTN and chronic illnesses have been strongly associated with progression of WMHs. On the contrary, early treatment with anti-hypertensives have been found to have a protective role in reducing both blood pressure and WMH progression [7]. Despite the role of HTN in COFP is not completely understood, in a recent case-control study including 242 BMS patients and an equal number of pain-free controls matched for age and gender, authors found a significant higher prevalence of HTN in BMS patients compared to controls (55.0% versus 33.5%) [8]. This finding may explain the potential contribution of HTN, in addition to other cardiovascular factors, to the higher prevalence of WMHs in patients affected by this subtype of COFP.

The causal role of pain on brain structure has been a matter of intense research during the last decades. Nowadays, data about brain functionality, especially considering white matter integrity suggest that chronic pain causes damage in its structure associated with a decrease in the brain neuroplasticity. Until now, the literature analysis is debated as it is difficult to understand if the chronic pain, including orofacial pain, may increase the risk of WMHs, or the early onset of WMHs may contribute later in the life in the development of chronic pain especially when WMHs are localized in the spinothalamic tract; probably it could be possible to consider that multiple comorbidities such as lifestyle behaviors, cardiovascular risk factors and chronic pain may act in a synergistic way contributing to the premature ageing of the brain that in turn may aggravate pain perception (Figure 1).

Therefore, considering the multifactorial pathophysiology of WMH burden and the complex interplay between cardiovascular and the nervous system it seems reasonable to include the assessment and treatment of vascular risk factors according with the treatment of orofacial pain condition in order to prevent and/or reverse the WMH onset and delay the neurodegenerative diseases progression.

Further studies should be performed to understand if a more careful evaluation and treatment of cardiovascular comorbidities, especially in ageing population, capable of improving brain neuroplasticity and avoiding the progression of WMHs may, in turn, contribute to reduce chronic pain symptoms.
